# Hepatic Encephalopathy in the 21st Century: Still an Emerging Topic

**DOI:** 10.3390/jcm10020298

**Published:** 2021-01-15

**Authors:** Lorenzo Ridola, Oliviero Riggio

**Affiliations:** Department of Translational and Precision Medicine, “Sapienza” University of Rome, viale dell’Università 37, 00185 Rome, Italy; oliviero.riggio@uniroma1.it

Why write about hepatic encephalopathy (HE) in the twenty-first century? It is in front of this question that we found ourselves, once invited to serve as guest editors for the Special Issue of the Journal of Clinical Medicine entitled “Hepatic Encephalopathy: Clinical Challenges and Opportunities”. Scientific research has made important advances, the pathogenesis of HE is more deeply known, and the role of toxins such as ammonia (still relevant!), as well as those of systemic inflammation, are being discussed, even in a very heated way. Overt HE has been deeply defined, while the Minimal Hepatic Encephalopathy (MHE) (and covert) are becoming increasingly important, due to the high impact on the daily life both of the patient and caregiver. Progress is also remarkable regarding the pharmacological approach. In fact, secondary prophylaxis appears to be consolidated and effective, while for episodic HE, the importance of identifying and treating precipitating factors, as an “ideal first cause” of HE, is now affirmed and widely accepted [1,2,3]. Nonetheless, there are still many gray areas and issues that need further study, especially regarding the role of treatment in patients with minimal HE [4,5,6]. In fact, in this specific setting, guidelines suggest treating patients on a case by case basis. More generally, the ideal design of therapeutic studies also remains debated. In fact, the existing literature on HE medical management still suffers from a lack of standardization, and this heterogeneity makes the pooling of data difficult or meaningless. There is still an unmet need for “robust” controlled clinical trials on treatment effects on HE, because decisive clinical studies are few, although the number of patients and their resource utilization remain high [6]. 

In this Special Issue, it was therefore decided to give space to contributions addressing the most innovative topics in HE. The pathogenesis of HE is, indeed, not completely clear. A series of observations, based on clinical and therapeutic features, suggest that for any substance to be involved in the pathogenesis of HE, it should originate in the gut, possibly by the action of bacteria, be found in the portal circulation, be increased in peripheral blood as a consequence of failing liver or portal systemic shunting, and finally should exert its effect on the brain. Unfortunately, any attempt to clarify the nature of the substance(s) involved has, until now, not been completely satisfactory. Ammonia meets all the above mentioned criteria, but its correlation with HE is not always found. Because of these considerations, the role of other potential toxins, such as tryptophan derivatives, has been investigated over the years [7] and the effects of systemic inflammation and its mediators have been better studied. However, even today, ammonia plays a predominant role in the pathogenesis of HE and this is also confirmed by the effectiveness of treatments aimed at ensuring the metabolism or the prompt elimination of nitrogen derivatives. There is growing interest in the modulation of the gut–liver–brain axis in the therapy of complications of liver cirrhosis, including HE. Fecal transplantation in this setting of patients has also recently been proposed with encouraging results [8,9]. This certainly represents a new frontier in the management of advanced liver diseases. Concerning HE pathophysiology, in this issue, the relationship between hepatic dysfunction and the progression of brain energy crisis in hepatic encephalopathy has also been addressed. 

Another unsolved problem, which still heavily complicates the management of cirrhotic patients with portal hypertension, is the occurrence of HE after transjugular intrahepatic portosystemic shunt (TIPS) placement. A TIPS is widely adopted to treat complications of portal hypertension such as recurrent variceal bleeding or refractory ascites, by shunting blood flow, bypassing the liver and, consequently, reducing portal pressure, with the aim to reduce mortality and bridge patients to liver transplant. TIPSs represent often a life-saving procedure, but are characterized, due to the blood diversion directly into systemic circulation, by the development of HE, particularly in the first period immediately after the procedure. To date, the role of drug therapy in prophylaxis of HE after TIPS is not yet clear and supported by strong scientific evidence [1,2,10]. More recently, approaches characterized by modulation of the stent caliber or choosing reduced caliber shunts have also been proposed [11,12,13,14]. However, even this evidence does not yet allow us to formulate sufficiently “robust” recommendations and the choice to close TIPSs for refractory HE is very hard and needs to be counterbalanced with the competing risk of newly developing those complications of portal hypertension that were the indication to TIPS placement. Recently, the role of spontaneous portosystemic shunts (SPSSs) also appears to be of growing interest, both for the causative role in determining HE, and due to the possibility to treat shunt-induced HE by closing or reducing the caliber of those collaterals [15,16,17,18]. Indeed, the search for SPSSs has entered the process of classification of patients with a history of HE. 

A frequent complication of cirrhosis is malnutrition, which is associated with the progression of liver failure, and with a higher rate of complications, including infections, hepatic encephalopathy, and ascites. It is well known that sarcopenia, a condition in which muscle mass and function are reduced due to the patient’s poor nutrition and reduced physical activity, also has a prognostic impact on cirrhosis and its complications [19]. A historical dietary approach of encephalopathic patients was characterized by a close protein restriction. Nevertheless, recently published European Association for the Study of the Liver guidelines on nutritional management of cirrhotic patients [20] state that nutritional status and the presence of sarcopenia should be evaluated in patients with HE and to avoid protein restriction in patients with HE. To date, the relationship between sarcopenia and HE looks better defined, as well as the importance of an adequate nutritional intake, but the role of an intervention deserves more convincing results. For example, studies aimed to assess the role of physical exercise in improving sarcopenia and consequently HE should, in our opinion, be strongly encouraged. 

In summary, HE is a condition characterized by a heavy burden both on the patient and caregiver, and on health systems. Therefore, considerable interest is currently focused on the use of administrative data. This information is available in databases of health care systems, and the retrieval and analysis of these data allow us not only to provide an immediate picture of the dimension of HE’s burden, but also to identify prognostic factors associated with the development of hepatic encephalopathy. This knowledge, in the near future, may allow for better stratification of patients at risk and starting early therapeutic interventions.

It is therefore time to consider HE under a new perspective, in which some different new factors should be considered to have a determinant/causative and prognostic role. Indeed, patients at risk for HE should be considered, not only those with severe liver disease, or a previous history or with minimal/covert HE, or TIPSs, but also those with sarcopenia, nutritional deficit, or bearing SPSSs. It is important to consider cirrhotic patients under this whole panorama in order to identify very high-risk patients, in which other factors with a different and non-“classical” management should be respectively searched and adopted.
